# Assessment of risk factors responsible for rapid deterioration of lung function over a period of one year in patients with chronic obstructive pulmonary disease

**DOI:** 10.1038/s41598-021-92968-5

**Published:** 2021-06-30

**Authors:** Anees ur Rehman, Shahid Shah, Ghulam Abbas, Sabariah Noor Harun, Sadia Shakeel, Rabia Hussain, Mohamed Azmi Ahmad Hassali, Muhammad Fawad Rasool

**Affiliations:** 1 Department of Pharmacy Practice, Faculty of Pharmacy, Bahauddin Zakariya University Multan, Multan, Pakistan; 2 Department of Clinical Pharmacy, School of Pharmaceutical Sciences, University Sains Malaysia, Gelugor, Malaysia; 3 Department of Pharmacy Practice, Faculty of Pharmaceutical Sciences, Government College University, Faisalabad, Faisalabad, Pakistan; 4 Department of Pharmaceutics, Faculty of Pharmaceutical Sciences, Government College University, Faisalabad, Faisalabad, Pakistan; 5 Department of Pharmacy Practice, Faculty of Pharmaceutical Sciences, Dow University of Health Sciences, Karachi, Pakistan; 6 Department of Social and Administrative Pharmacy, School of Pharmaceutical Sciences, University Sains Malaysia, Gelugor, Malaysia

**Keywords:** Diseases, Respiratory tract diseases, Risk factors

## Abstract

Compromised lung function is a common feature of COPD patients, but certain factors increase the rate of lung function decline in COPD patients. The objective of the current study was to investigate the effect of different clinically important factors responsible for rapid deterioration of lung function quantified as ≥ 60 ml decline in FEV1 over a period of one year. COPD patients recruited from the chest clinic of Penang hospital were followed-up for one year from August 2018 to August 2019. Rapid deterioration of lung function was defined as greater than 60 ml/year decline in force expiratory volume in one second. Among 367 included patients 73.84% were male, with mean age 65.26 (9.6) years and % predicted FEV_1_ 51.07 (11.84). 30.27% patients showed mean decline of ≥ 60 ml in FEV1. The regression analysis showed that current smoking relative risk (RR) = 2.38 (1.78–3.07), *p* < 0.001); GOLD Stage III& IV RR = 1.43 (1.27–1.97), *p* < 0.001); mMRC score 3 to 4 RR = 2.03 (1.74–2.70), *p* < 0.01); SGRQ-C score ≥ 10 points difference RR = 2.01 (1.58–2.73), *p* < 0.01); SGRQ-C symptoms Score ≥ 10 points difference RR = 1.48 (1.23–2.29), *p* < 0.001); 6MWT < 350 m RR = 2.29 (1.87–3.34), *p* < 0.01); ≥ 3 exacerbation in study year RR = 2.28 (1.58–2.42, *p* < 0.001); 8 or more hospital admission days (RR = 3.62 (2.66–4.20), *p* < 0.001); Charlson comorbidity index ≥ 3 RR = 3.18 (2.23–3.76), *p* < 0.01) and emphysema RR = 1.31 (1.15–1.79), *p* < 0.01) were significant risk factors for the rapid deterioration of lung function (FEV1 decline ≥ 60 ml). Among different factors CCI score ≥ 3, abrupt decline in health status, exacerbation frequency ≥ 3, hospital admission days ≥ 8 and emphysema were reported as risk factors for rapid deterioration of lung function.

## Introduction

Chronic obstructive pulmonary disease (COPD) is associated with substantial economic burden and is among the leading causes of morbidity and mortality worldwide^1^. It is affecting more than 300 million word’s population and is responsible for 3 million deaths every year worldwide^2^. Spirometry test quantified as forced expiratory volume in 1 second (FEV_1_) is a GOLD (Global initiative for chronic obstructive lung disease) recommended COPD diagnostic test and is one of the most reliable and reproducible measurements of airflow obstruction^3^. Current diagnosis system relies on it as an indicator of disease progression, categorize COPD patients on the basis of disease severity, delineate risk factors, and lung function impairment under the influence of COPD^3^. The European Medicines Agency recommend to use the change in FEV_1_ value during baseline and follow-up as an indicator of efficacy of therapeutic interventions in COPD clinical trials^4^.

Compromised lung function is a common feature of COPD patients, but certain factors increase the rate of lung function decline in COPD patients. Excessive decline in lung function is associated with increased exacerbation, premature mortality and compromised quality of life in COPD patients^5^. Research shows that pharmacological therapies and interventions show more efficacy in patients with higher FEV_1_ than in patients with lower FEV_1_^6^. Slowing down the progression of disease is an achievable goal in management of COPD^3^. Initially smoking was considered as primary cause for progression of COPD and quitting smoking was considered as most important preventive measure to slow down progression of disease^7^. Research shows smoking is not the only risk factor and quitting smoking is not the only preventive measure to reduce progression of disease^8^. Exposure to biomass fuel burning, pollution, atopy, alpha-1 antitrypsin deficiency, antioxidant deficiency, respiratory-tract infections, airway inflammation, asthma, emphysema, chronic bronchitis, and poor socioeconomic status are also potential risk factors for initiation and progression of COPD^6,9,10^.

Identification of factors responsible for rapid deterioration of lung function in COPD patients, has important public health implications. The ability to accurately identify patients at increased risk of deterioration in lung function and rapid progression of disease would help to inhibit lung function deterioration and limit disease in acute phase by taking in time measures. Knowledge about factors responsible for rapid progression of disease can also play an important role in disease management decisions and initiation or withdrawal of a therapy. This system of prognosis of disease proved its effectiveness in management of community-acquired pneumonia, where implementation of CURB-65 tool in clinical practice resulted in improved patient care^11^. Similar prognostic method in COPD to identify the risk factors responsible for rapid deterioration of lung function, can help to direct resources to the ones in need, reduce progression of disease, reduce exacerbation, improve patient care and reduced burden on healthcare system^12^.

However, the evidence of such factors on rapid deterioration of lung function is limited due to unavailability of sufficient number of studies with repeated lung function measurements during specific time period. Previous studies have examined the risk factors responsible for decline in FEV_1_ value in COPD patients, but they were performed in specific populations, such as patients suffering from severe COPD or frequent exacerbations^8,13,14^. Few studies assessed the impact of specific factors on FEV_1_ decline, such as specific therapies^15^, emphysema or chronic bronchitis^16^, exercise capacity^17^, Occupational exposures^18^, biomass fuel^19^, and clinical variables^12^. There is a lack of consistency in identified factors due to difference in study design, inclusion criteria, study duration and parameters investigated. Given the discrepancies between and limitations of the studies conducted to date, we investigated the effect of different clinically important factors responsible for rapid deterioration of lung function quantified as ≥ 60 ml decline in FEV_1_ over a period of one year in a single study. Addition of different potential factors in a multivariate analysis model gives true impact of a factor in presence of different factors.

## Methodology

### Study design and participants

This was a longitudinal prospective cohort. Sample size was calculated using Daniel formula on the basis of prevalence of disease^20^. The calculated sample size including 20% dropout was 367 COPD patients. Patients were recruited at the at chest clinic of Penang hospital. Patients were included if (1) they have confirmed diagnosis of COPD according to GOLD guidelines (FEV_1_/FVC ratio < 70%), (2) age ≥ 40 years, (3) no exacerbation in last 4 months, (5) enrolled in the chest clinic and having medical history of at least one year. Patients were excluded from the study if (1) they undergone through lung surgery or lung transplantation, (2) participating in any pulmonary rehabilitation program (PR), (3) inability to complete pulmonary function test and (4) inability to compete the questionnaire. Severity of COPD was defined based on GOLD 2018 classification. Ethical approval for this study was obtained from the Medical Research and Ethics Committee (MREC), Ministry of Health Malaysia (Registration number: NMRR-18-1482-42075). This study was conducted in accordance with the Declaration of Helsinki. Written informed consent was obtained from all participants.

### Clinical data and measured outcome

Data was collected from all included patients using a self-administered standardized questionnaire. The information obtained included social and demographic data, respiratory symptoms, smoking history, workplace history, previous asthma history, years with COPD, exacerbation history, risk factors, preventive care (influenza and pneumonia vaccination), comorbidities with Charlson Comorbidity Index (CCI), exercise capacity by six-minute walk distance test (6MWT), modified medical research council dyspnoea scale (mMRC), COPD assessment test (CAT), St. George’s Respiratory COPD specific Questionnaire (SGRQ-C) scale and pharmacological treatment during management phase and exacerbation phase.

No instructions regarding treatment were provided to the healthcare professionals and treatment was decided solely by patient’s physician. Exacerbation was defined as worsening of COPD symptoms requiring emergency department visit or hospital admission. The frequency of exacerbation was calculated during follow-up period. Patients were categorized as suffering from 1, 2, 3 or more than 3 exacerbations during study period. Severity of exacerbation was defined as hospital admission for more than 7 days or as judged by the physician. Data on comorbidities were collected from patient’s medical files. CCI was used to measure the impact of comorbidities. CCI is a standardized scale with 15 chronic diseases, graded according to the severity of disease^21^.

Patients recruited into the study were then followed up for one year from August 2018 to August 2019. The procedures were repeated every 4th month during the follow-up. The yearly visits data is presented here. Data recorded at the 4th, and 8th month visits have not been analyzed in this study as data on some patient characteristics were not available at these time points.

Spirometry was performed according to American thoracic society guidelines^22^. Spirometry was performed 45 min after bronchodilation with 400 μg salbutamol via a spacer. The mean difference in FEV_1_ was reported as difference in baseline visit and yearly visit. In COPD patients different thresholds of ≥ 40 mL/year and ≥ 60 ml/year^8,19^, have been used in literature to define rapid decline in FEV_1_. For the purpose of this study, decline in FEV_1_ ≥ 60 ml/year was considered as rapid decline because it is significant and clinically important according to ATS/ERS recommendations^23^. Severity of COPD was categorised according to spirometry results, in accordance with GOLD 2018 guidelines^3^. Grade I COPD with FEV_1_ ≥ 80% predicted, grade II with FEV_1_ 50% to 80% predicted, grade III with FEV_1_ 30% to 50% predicted, and grade IV with FEV_1_ < 30% predicted.

Sputum analysis was done at the pathology lab of Penang hospital to assess the presence of inflammation. A sputum eosinophil percentage ≥ 2.5% was used to define eosinophilic inflammation^24^. Chronic bronchitis was defined by the presence of chronic cough and sputum production for three consecutive months in two consecutive years. Computed tomography scan (CT scan) were done at the imaging unit of Penang hospital to distinguish emphysematous regions from non-emphysematous gas trapping regions. The reports were evaluated by the radiologist to confirm the presence or absence of emphysema. The presence of emphysema on CT scan was defined as well demarcated areas of decreased attenuation, as compared with contiguous normal lung tissue. The whole lung was divided into six zones (left and right zones in the upper, middle and lower lung fields). Low attenuation areas (LAA) in each image section were scored on a scale of 0 to 4, where 0 = no LAA, 1 = 1–25%, 2 = 26–50%, 3 = 51–75%, and 4 = 76–100% LAA. Grades for the images of each zone were added to yield the total LAA score.

For 6MWT, patients were requested to walk as far as possible at their own pace in 6 min in long hospital corridor (48.76 m long) adjacent to the chest clinic. The distance covered was recorded in meters. Patients were allowed to stop and rest during the test, but were instructed to resume walking as soon as they felt able to do so. The test was supervised by a well-trained researcher according to the ATS guidelines^25^.

### Health status measures

Malaysian version of SGRQ-C and mMRC dyspnea scale were used to collect data about health status^26,27^. SGRQ-C is a self-administered, disease specific, COPD questionnaire comprising symptoms, activity and impact subscale. Each subscale score and total score range from 0 to 100, with 100 shows the worst quality of life. For SGRQ-C a threshold of 10 points increase from baseline is considered as clinically significant deterioration in health status^28^. Therefore, to assess the relationship of SGRQ-C scores and rate of lung function decline, the difference in SGRQ-C scores between follow-up and baseline were categorized as ≤ 10 points difference and > 10 points difference.

CAT is an FDA approved health status questionnaire used for assessment of COPD patients. CAT is easy to understand and consists of 8 items related to symptoms and activities. Each item has scores 0 to 5 from best to worst with a maximum total score of 40^29^.

MMRC dyspnoea scale is a 5-item WHO recommended scale to assess the degree of breathlessness in COPD patients^26^. It is widely used in clinical practice and used to categorise COPD patients based on symptoms burden as recommended in 2019 GOLD guidelines^3^.

### Analysis

Frequencies and percentages were reported for categorical variables and the mean and standard deviation for continuous variables. Normality of the data was checked using Kolmogorov–Smirnov test and Shapiro–Wilk test. Characteristics of patients in different groups stratified according to FEV_1_ decline were compared using independent-samples t-test for continuous variables and Chi-square test for categorical variables. Univariate and multivariate Cox regression with robust variance were performed to estimate relative risk (RR) for different factors i.e. body mass index (BMI), smoking status (ex or current smoker), age, disease severity, degree of dyspnoea exercise capacity, difference in health status over a period of one year (SGRQ-C total, symptoms, activity and impact scores), exacerbation frequency (0, 1, 2, 3, or > 3), hospital admission days, CCI scores, inflammation, LABA (long-acting beta agonist) use, LAMA (long acting muscarinic antagonist) use, vaccination, and occupational hazards on decline in FEV_1_ > 60 ml/year after one year of follow up. First a univariate analysis was performed to assess important factors^30^. Factors having substantial impact (*p* values < 0.20) were added in multivariate model. The same analysis was again performed on a subset of 109 patients showing ≥ 60 ml decline in FEV1 over a period of one year to assess the impact of above-mentioned factors on rapid decline of FEV1. Because the amount of missing data was only 2.3%, a complete case analysis was performed^30^. A *p* value of < 0.01 was considered to be statistically significant. An adjustment for multiple testing was made with a Bonferroni correction when applicable. All analysis were performed using STATA software (https://www.stata.com/; StataCorp. 2009. Stata 11 Base Reference Manual. College Station, TX: Stata Press).

## Results

A total of 367 patients were enrolled in the study during baseline. Most of the patients were male 271 (73.84%), with mean age 65.26 (9.6) years and % predicted FEV_1_ 51.07 (11.84). During baseline 175 (47.68%) patients were suffering from moderate COPD (GOLD Stage I&II) and 192 (52.32%) patients were suffering from severe COPD (GOLD Stage III& IV). Mean CCI score was reported as 2.03 (1.44). When stratified according to decline in FEV_1_ significant difference was observed in BMI, current smoking, severity of disease, mMRC score, CAT Score, SGRQ–C scores, distance covered in 6MWT and CCI among the patients with ≤ 60 ml decline in FEV_1_ and ≥ 60 ml decline in FEV_1_. Baseline demographic and clinical data of COPD patients stratified according to decline in FEV_1_ are presented in Table 1.

**Table 1:** Baseline demographic and clinical data of COPD patients included in the study.

Variables	Overall COPD patients (367)	≤ 60 ml decline in FEV_1_ (253)	≥ 60 ml decline in FEV_1_ (114)	*p* value ^a^
No. of patients	367 (100%)	253 (68.93%)	114 (31.06%)	–
Age	65.26 (9.6)	64.38 (9.5)	67.21 (9.5)	0.37
Male	271 (73.84%)	189 (73.25%)	82 (71.92%)	0.65
BMI	24.23 (4.27)	24.85 (4.21)	22.04 (3.89)	< 0.01
Years with COPD	7.2 (6.1)	6.3 (5.6)	9.3 (6.4)	0.31
Smoking status				
Current smokers	68 (18.89%)	29 (11.46%)	39 (34.21%)	< 0.001
Ex-smokers	301 (82.01%)	209 (82.60%)	92 (80.70%)	0.26
Post-bronchodilator spirometry				
FEV_1_%	51.07 (11.84)	56.67 (11.54)	43.41 (11.19)	< 0.01
FEV_1_ (L)	1.38 (0.54)	1.56 (0.51)	1.14 (0.60)	< 0.01
GOLD Stage I & II	175 (47.68%)	119 (47.03%)	56 (49.12%)	0.29
GOLD Stage III & IV	192 (52.32%)	134 (52.97%)	58 (50.88%)	< 0.001
Medication				
LABA	236 (64.31%)	183 (72.33%)	53(46.49%)	< 0.01
LAMA	191 (52.04%)	130 (68.06%)	61 (53.51%)	0.12
Health status measures				
mMRC dyspnea	2.76 (0.8)	2.37 (0.8)	3.30 (1.10)	< 0.001
CAT Score	20.05 (7.62)	17.43 (6.75)	26.68 (7.12)	< 0.01
SGRQ-C Total	46.48 (28.61)	40.66 (35.48)	59.30 (36.65)	< 0.001
SGRQ-C Symptom Score	51.46 (29.48)	46.23 (25.95)	58.70 (29.33)	< 0.001
SGRQ-C Activity Score	45.91 (28.17)	44.42 (26.5)	51.4 (29.53)	< 0.01
SGRQ-C Impact Score	40.47 (32.78)	38.87 (29.72	48.07 (27.24)	0.07
6MWT in meters	429.8 (84.32)	484.2 (73.45)	339.4 (87.14)	< 0.001
Comorbidities				
CCI	2.03 (1.44)	1.47 (0.92)	2.9 (1.34)	< 0.01

Data are presented as n (%) and mean (SD) unless otherwise stated.

BMI, body mass index; COPD, Chronic Obstructive Pulmonary Disease; CAT, COPD assessment test; CCI, Charlson comorbidity Index; FEV_1_, forced expiratory volume in 1 second; FVC, forced vital capacity; FEV_1_%, percentage predicted FEV1; GOLD, global initiative for Chronic Obstructive Lung Disease; GOLD Stage I (FEV1 ≥ 80% predicted); GOLD stage II (50% ≤ FEV1 < 80% predicted); GOLD Stage III (30% ≤ FEV1 < 50% predicted); GOLD Stage IV (FEV1 < 30% predicted); LABA, long-acting beta agonist; LAMA, long acting muscarinic antagonist; 6MWT, six minute walking distance travelled; mMRC, modified medical research council dyspnoea scale, SGRQ-C, St George’s Respiratory COPD specific Questionnaire.

^a^The difference was assessed among groups stratified according to decline in FEV_1_.

### Longitudinal assessment

**Figure 1:**
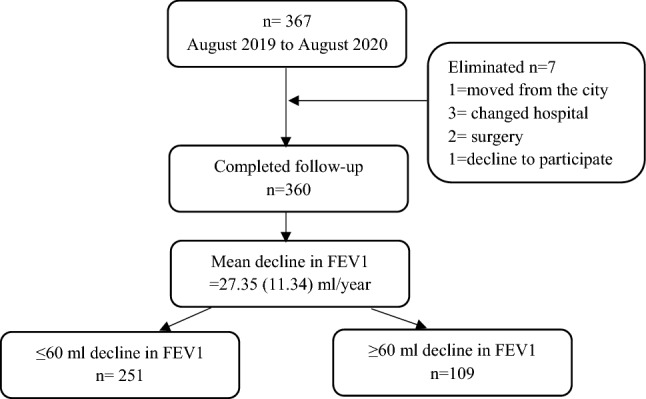
Consort diagram of the study population.

Three hundred and sixty patients completed the follow-up. Seven patients lost the follow-up due to different reasons (Fig. 1).

**Figure 2:**
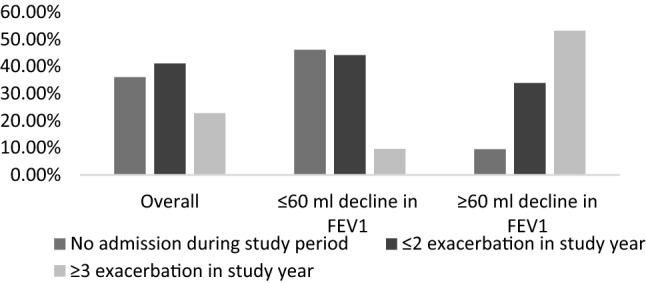
Frequency of exacerbation stratified according to FEV1 decline.

**Figure 3:**
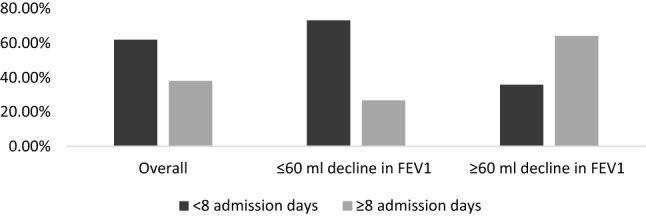
Hospital admission days stratified according to FEV1 decline.

During the study period mean decline in FEV_1_ was observed as 27.35 (11.34) ml. Among the included patients 251 (69.72%) patients showed mean decline of ≤ 60 ml, while 109(30.27%) patients showed mean decline of ≥ 60 ml in FEV1. Smoking cessation was observed in 17(4.72%) patients. 31(8.3%) patients moved from moderate COPD to severe COPD at the end of the study year. Emphysema and chronic bronchitis were present in 198 (55%) and 220 (61.11%) patients. During the follow-up 130 (36.11%) patients didn’t suffer to an exacerbation, 148 (41.11%) patients suffered ≤ 2 exacerbation and 82 (22.77%) patients suffered ≥ 3 exacerbation (Figs. 2 and 3).

Demographic and clinical characteristics of COPD patients stratified according to decline in FEV_1_ after one year of follow-up are presented in Table 2.

**Table 2:** Demographic and clinical data of COPD patients included in the study after one year follow-up.

Variables	Overall	≤ 60 ml decline in FEV_1_ (251)	≥ 60 ml decline in FEV_1_ (109)	*p* value ^a^
No. of patients	360 (100%)	251 (69.72%)	109 (30.27%)	–
BMI	23.97 (4.19)	24.53 (5.2)	21.62 (4.26)	< 0.01
Current smokers	51 (14.16%)	24 (9.56%)	27 (28.44%)	< 0.001
Post-bronchodilator spirometry				
FEV_1_%	51.03 (10.21)	54.7 (10.43)	37.36 (10.75)	< 0.01
FEV_1_ (L)	1.35 (0.66)	1.51 (0.32)	1.06 (0.42)	< 0.01
GOLD Stage I & II	142 (39.44%)	111 (44.22%)	31 (28.44%)	0.21
GOLD Stage III & IV	218 (60.56%)	140 (55.77%)	78 (71.55%)	< 0.001
Health status measures				
mMRC dyspnea	2.92 (0.8)	2.42	3.87 (0.9)	< 0.001
CAT Score	22.71 (6.88)	18.46 (7.62)	29.96 (7.37)	< 0.01
SGRQ-C Total	52.23 (26.44)	44.26 (25.54)	68.20 (26.12)	< 0.001
SGRQ-C Symptom Score	53.4 (28.32)	51.35 (23.42)	67.45 (30.28)	< 0.001
SGRQ-C Activity Score	48.69 (26.43)	45.97 (26.5	59.41 (26.42)	< 0.01
SGRQ-C Impact Score	42.37 (30.82)	39.36 (28.34)	57.39 (27.21)	< 0.01
6MWT in meters	418.5 (80.28)	481.8 (76.42)	323.3 (78.40)	< 0.001
6MWT > 350 m	221 (61.39%)	193 (76.89%)	28 (25.69%)	
6MWT < 350 m	139 (38.61%)	58 (23.10%)	81 (74.31%)	
Medication				
LABA	242 (67.22%)	189 (75.30%)	53(48.62%)	< 0.01
LAMA	186 (52.04%)	131 (52.19%)	55 (50.46%)	0.17
Comorbidity Index				
CCI	2.12 (1.44)	1.49 (0.92)	3.1 (1.34)	< 0.001
CCI < 3	255 (69.48%)	219 (86.56%)	36 (31.58%)	
CCI ≥ 3	112 (30.51%)	34 (13.44%)	78 (68.42%)	
Emphysema	198 (55%)	125 (49.80)	73 (66.97%)	< 0.01
Chronic bronchitis	220 (61.11%)	133 (52.99%)	87 (79.82%)	< 0.01
Exacerbation				
Exacerbation per year	1.20 (1.4)	0.91 (1.1)	2.6 (1.7)	< 0.01
No admission during study period	130 (36.11%)	116 (46.21%)	14 (9.47%)	0.19
≤ 2 exacerbation in study year	148 (41.11%)	111 (44.22%)	37 (33.95%	< 0.001
≥ 3 exacerbation in study year	82 (22.77%)	24 (9.56%)	58 (53.21%)	< 0.001
Hospital stay days	4.9 (4.8)	4.5 (4.2)	7.2 (5.3)	< 0.001
< 8 admission days	223 (61.94%)	184 (73.30%)	39 (35.78%)	
≥ 8 admission days	137 (38.06%)	67 (26.69%)	70 (64.22%)	
Eosinophilic inflammation				
Eosinophil count %	1.7% (1–2.8)	1.7% (1.1–2.8)	1.8% (1.1–2.1)	0.12
Eosinophil count ≥ 3%	141 (31.67%)	62 (24.70%)	52 (47.60%)	
Vaccination	68 (18.89%)	57 (22.71%)	11 (10.09%)	< 0.01
Occupational hazards	48 (13.33)	31 (12.35%)	17 (15.60%)	< 0.01

Data are presented as n (%) and mean (SD) unless otherwise stated.

^a^The difference was assessed among groups stratified according to decline in FEV1.

BMI, body mass index; COPD, Chronic Obstructive Pulmonary Disease; CAT, COPD assessment test; CCI, Charlson comorbidity Index; FEV_1_, forced expiratory volume in 1 second; FVC, forced vital capacity; FEV_1_%, percentage predicted FEV_1_; GOLD, global initiative for Chronic Obstructive Lung Disease; GOLD Stage I (FEV1 ≥ 80% predicted); GOLD stage II (50% ≤ FEV1 < 80% predicted); GOLD Stage III (30% ≤ FEV1 < 50% predicted); GOLD Stage IV (FEV1 < 30% predicted); LABA, long-acting beta agonist; LAMA, long acting muscarinic antagonist; 6MWT, six minute walking distance travelled; mMRC, modified medical research council dyspnoea scale, SGRQ-C, St George’s Respiratory COPD specific Questionnaire.

After one year of follow-up statistically significant difference was observed in mMRC score (*p* < 0.01), CAT Score (*p* < 0.001), SGRQ-C total Score (*p* < 0.001) and SGRQ-C symptom score (*p* < 0.01) as compared to their baseline values. Mean change in health status scores after one year from baseline is represented in Table 3.

**Table 3:** Change in health status measures scores from baseline to one year follow-up.

Health status measures	Baseline values	One year follow-up values	Mean difference	*p* value
mMRC	2.76 (0.8)	2.92 (0.8)	0.16 (0.04)	< 0.01
CAT Score	20.05 (7.62)	22.71 (6.88)	2.66 (1.2)	< 0.001
SGRQ-C Total	46.48 (28.61)	52.83 (26.44)	6.35 (4.53)	< 0.001
SGRQ-C Symptom Score	50.46 (29.48)	55.4 (28.32)	4.94 (3.61)	< 0.01
SGRQ-C Activity Score	44.91 (28.17)	50.69 (26.43)	5.78 (3.50)	0.03
SGRQ-C Impact Score	39.11 (32.78)	43.77 (30.82)	4.66 (7.61)	0.19
6MWT in meters	429.8 (84.32)	418.5 (80.28)	− 11.3	< 0.01

Data are presented as mean (SD) unless otherwise stated.

COPD, Chronic Obstructive Pulmonary Disease; mMRC, modified medical research council dyspnoea scale; CAT, COPD assessment test; SGRQ-C, St George’s Respiratory COPD specific Questionnaire.

### Risk factors influencing ≤ 60 ml decline in FEV_1_ in COPD patients

After one year of follow-up 251 patients showed ≤ 60 ml decline in FEV_1_. Table 4 shows the risk associated with different factors for ≤ 60 ml decline in FEV1. The multivariate regression analysis showed that age RR = 1.12 (0.79–1.34), *p* < 0.01; current Smokers RR = 3.27 (2.56–3.34), *p* < 0.001; GOLD Stage III& IV RR = 3.48 (2.72–4.36), *p* < 0.001; mMRC score 2 to 3 RR = 2.17 (1.64–2.88), *p* < 0.01; mMRC score 3 to 4 RR = 2.54 (2.13–3.20), *p* < 0.001; SGRQ-C score ≥ 10 points difference RR = 3.27 (2.78–3.91), *p* < 0.001; SGRQ-C symptoms Score ≥ 10 points difference RR 2.32 (1.93–3.18), *p* < 0.001; SGRQ-C activity Score ≥ 10 points difference RR = 1.74 (1.79–2.80), *p* < 0.001; 6MWT < 350 m RR = 2.69 (2.19–3.84), *p* < 0.001; ≤ 2 exacerbation in study year RR = 1.54 (1.15–1.84), *p* < 0.01; ≥ 3 exacerbation in study year RR = 3.49 (2.85–3.73), *p* < 0.001; 8 or more hospital admission days RR = 3.91 (3.36–4.30), *p* < 0.001; CCI score ≤ 3 RR = 1.49 (1.14–1.72), *p* < 0.01; CCI ≥ 3 RR = 3.46 (2.73–4.13), *p* < 0.001; emphysema RR = 1.24 (1.03–1.52), *p* < 0.01; chronic bronchitis RR = 1.12 (0.86–1.35), *p* < 0.01; eosinophil count ≥ 3% RR = 1.78 (0.82–2.12), *p* < 0.001; and occupational Hazards RR = 1.43 (1.14–1.88), *p* < 0.01 were significant risk factors for normal deterioration of lung function (FEV_1_ decline ≤ 60 ml) in COPD patients.

**Table 4:** Multiple logistic regression models to assess the risk factors associated with ≤ 60 ml decline in FEV1 in COPD patients.

Risk factors	≤ 60 ml decline in FEV1 (251)		
	RR	95% CI	Sig
BMI	1.21	0.85–1.31	0.11
Age	1.12	0.79–1.34	0.01
Gender	0.73	0.29–1.04	0.08
Smoking status			
Current smokers	3.27	2.56–3.34	< 0.001
Disease severity			
GOLD Stage I & II	0.74	0.47–1.13	0.21
GOLD Stage III & IV	3.48	2.72–4.36	< 0.001
mMRC Dyspnea			
mMRC score 2 to 3	2.17	1.64–2.88	< 0.01
mMRC score 3 to 4	2.54	2.13–3.20	< 0.001
Difference in health status measure Scores over a period of one year			
SGRQ-C score ≥ 10 points difference	3.27	2.78–3.91	< 0.001
SGRQ-C symptoms Score ≥ 10 points difference	2.32	1.93–3.18	< 0.001
SGRQ-C activity Score ≥ 10 points difference	1.74	1.79–2.80	< 0.001
Exercise capacity			
6MWT > 350 m	1.13	0.81–1.29	0.07
6MWT < 350 m	2.69	2.19–3.84	< 0.001
Exacerbation			
≤ 2 exacerbation in study year	1.54	1.15–1.84	0.01
≥ 3 exacerbation in study year	3.49	2.85–3.73	< 0.001
Hospital admission days			
1 to 7	1.31	0.91–1.49	0.05
8 or more	3.91	3.36–4.30	< 0.001
Comorbidities			
CCI ≤ 3	1.49	1.14–1.72	< 0.01
CCI ≥ 3	3.46	2.73–4.13	< 0.001
Emphysema	1.24	1.03–1.52	< 0.01
Chronic Bronchitis	1.12	0.86–1.35	< 0.01
Inflammation			
Eosinophil count ≥ 3%	1.78	0.82–2.12	< 0.001
Vaccination	0.53	0.27–0.69	0.42
Medication			
LABA	0.72	(0.64–0.88)	< 0.01
LAMA	0.79	(0.62–0.92)	< 0.01
Occupational Hazards	1.43	1.14–1.88	< 0.01

BMI, body mass index; CI, confidence interval; COPD, Chronic Obstructive Pulmonary Disease; CCI, Charlson comorbidity Index; FEV_1_, forced expiratory volume in 1 second; FVC, forced vital capacity; FEV_1_%, percentage predicted FEV1; GOLD, global initiative for Chronic Obstructive Lung Disease; GOLD I (FEV_1_% > 80); GOLD II (FEV_1_% 50–79); GOLD III (FEV_1_% 30–49); GOLD IV (FEV_1_% < 30); mMRC, modified medical research council dyspnoea scale, SGRQ-C, St George’s Respiratory COPD specific Questionnaire; LABA, long-acting beta agonist; LAMA, long acting muscarinic antagonist; RR, Relative Risk; spirometry was post bronchodilator; Vaccination, Pneumonia and influenza vaccination; 6MWT, six minute walking distance test.

### Risk factors influencing the rapid lung function deterioration (≥ 60 ml decline in FEV_1_) in COPD patients

After one year follow-up 109 patients showed rapid deterioration in lung function (≥ 60 ml decline in FEV_1_). Table 5 shows the risk associated with different factors for ≥ 60 ml decline in FEV_1_. The regression analysis showed that current smoking RR = 2.38 (1.78–3.07), *p* < 0.001; GOLD Stage III& IV RR = 1.43 (1.27–1.97), *p* < 0.001; mMRC score 3 to 4 RR = 2.03 (1.74–2.70), *p* < 0.01; SGRQ-C score ≥ 10 points difference RR = 2.01 (1.58–2.73), *p* < 0.01; SGRQ-C symptoms Score ≥ 10 points difference RR = 1.48 (1.23–2.29), *p* < 0.001; 6MWT < 350 m RR = 2.29 (1.87–3.34), *p* < 0.01; ≥ 3 exacerbation in study year RR = 2.28 (1.58–2.42, *p* < 0.001; 8 or more hospital admission days RR = 3.62 (2.66–4.20), *p* < 0.001; CCI ≥ 3 RR = 3.18 (2.23–3.76), *p* < 0.01 and emphysema RR = 1.31 (1.15–1.79), *p* < 0.01 were significant risk factors for the rapid deterioration of lung function (FEV_1_ decline ≥ 60 ml).

**Table 5:** Multiple logistic regression models to assess the risk factors associated with ≥ 60 ml decline in FEV_1_ in COPD patients.

Risk factors	≥ 60 ml decline in FEV1 (109)		
	RR	95% CI	Sig
BMI	1.15	0.73–1.35	0.17
Age	0.74	0.35–1.01	0.36
Gender	0.67	0.37–0.97	0.14
Smoking status			
Current smokers	2.38	1.78–3.07	< 0.001
Disease severity			
GOLD Stage I & II	0.87	0.61–1.25	0.42
GOLD Stage III & IV	1.43	1.27–1.97	< 0.001
mMRC dyspnea			
mMRC score 2 to 3	1.36	1.17–1.82	0.22
mMRC score 3 to 4	2.03	1.74–2.70	< 0.01
Difference in health status measure Scores over a period of one year			
SGRQ-C score ≥ 10 points difference	2.01	1.58–2.73	< 0.01
SGRQ-C symptoms Score ≥ 10 points difference	1.48	1.23–2.29	< 0.001
SGRQ-C activity Score ≥ 10 points difference	1.15	1.01–1.48	0.22
Exercise capacity			
6MWT > 350 m	0.81	0.21–0.97	0.53
6MWT < 350 m	2.29	1.87–3.34	< 0.01
Exacerbation			
≤ 2 exacerbation in study year	1.07	0.61–1.44	0.18
≥ 3 exacerbation in study year	2.28	1.58–2.42	< 0.001
Hospital admission days			
1 to 7	1.04	0.53–1.32	0.23
8 or more	3.62	2.66–4.20	< 0.001
Comorbidities			
CCI ≤ 3	1.12	0.46–1.42	0.30
CCI ≥ 3	3.18	2.23–3.76	< 0.01
Emphysema	1.31	1.15–1.79	< 0.01
Chronic Bronchitis	1.18	0.77–1.45	0.05
Inflammation			
Eosinophil count ≥ 3%	1.39	0.82–2.12	< 0.001
Vaccination	0.67	0.29–0.86	0.32
Medication			
LABA	0.75	(0.64–0.86)	0.03
LAMA	0.82	(0.72–1.09)	0.15
Occupational hazards	1.21	0.73–1.47	0.10

BMI, body mass index; CI, confidence interval; COPD, Chronic Obstructive Pulmonary Disease; CCI, Charlson comorbidity Index; FEV_1_, forced expiratory volume in 1 second; FVC, forced vital capacity; FEV_1_%, percentage predicted FEV1; GOLD, global initiative for Chronic Obstructive Lung Disease; GOLD I (FEV_1_% > 80); GOLD II (FEV_1_% 50–79); GOLD III (FEV_1_% 30–49); GOLD IV (FEV_1_% < 30); mMRC, modified medical research council dyspnoea scale, SGRQ-C, St George’s Respiratory COPD specific Questionnaire; LABA, long-acting beta agonist; LAMA, long acting muscarinic antagonist; RR, Relative Risk; spirometry was post bronchodilator; Vaccination, Pneumonia and influenza vaccination; 6MWT, six minute walking distance test.

## Discussion

In COPD management multidimensional assessment is an established strategy for better assessment of patients. But it is still confusing which factors are best predictor for rapid deterioration of lung function. This study reports impact of different factors on rapid deterioration of lung function in COPD patients. current smoking, disease severity, mMRC score 3 to 4, SGRQ-C score ≥ 10 points difference, SGRQ-C symptom score ≥ 10 points difference, 6MWT < 350 m, ≥ 3 exacerbation per year, 8 or more hospital admission days, CCI ≥ 3 and emphysema were significantly associated with decline in FEV_1_ ≥ 60 ml/year. During the study year mean annual decline in FEV_1_ was observed as 27.35 (11.34) ml/year, whereas, 30.27% patients showed more than 60 ml/year decline in FEV_1_. This is quite close to a previously reported study in which they reported that one third of the patients showed a rapid decline of 78 ml/ year in FEV_1_ value^31^. Another study reported mean decline of 66 ml/year in FEV_1_ in patients with rapidly declining FEV_1_ as compared to mean decline of 28 ml/year in rest of the patients^8^.

Health status of COPD patients was assessed using SGRQ-C questionnaire. The rate of progression in lung function decline increased with increase in the difference between baseline and follow-up scores. Patients who reported greater difference in baseline and follow-up SGRQ-C scores showed rapid deterioration of lung function. The rate was significant when patients showed ≥ 10 points difference in total and symptom score over a period of one year. Our results are in line with the study of Alma et al. who reported that, a difference of 10 points in SGRQ-C score was associated with significant deterioration in health status of COPD patients^28^. Our results strengthen the fact that deterioration in health status is associated with rapid decline in lung function.

Severity of Dyspnea was assessed using mMRC dyspnea scale. MMRC dyspnea scale is a well-established tool to assess the severity of dyspnea. Higher score indicates higher level of breathlessness^26^. Higher dyspnea score was significantly related to rapid deterioration of lung function. In fact, it was one of the strongest risk factors for FEV_1_ decline. Dyspnoea is associated with physiological, cognitive, psychological and emotional responses. These responses may stimulate the rapid deterioration of lung function. In a large prospective study dysponea was reported to be a better predictor of mortality than the BODE index^32^. Thus, controlling dyspnea can slow down the progression of COPD. Different therapeutic interventions such as desensitization, relaxation and breathing retraining, monitored activity training and self-management can help to minimize the effects of dysponea and limit the rate of lung function decline^33^. Severity of disease showed significant impact on the progression of disease. People in GOLD grade I and II didn’t showed significant decline in lung function. Patients with severe airway obstruction were prone to rapid disease progression. This may be due to the fact that in severe airway obstruction, lungs have reduced respiratory reserves and decompensate in response to small triggers^11^. GOLD III and GOLD IV patients experience severe level of dyspnoea. Dyspnoea, causes forceful berating and utilization of energy to breath. This causes load on lungs and results in rapid destruction of airways^32^.

Emphysema and chronic bronchitis are important clinical manifestations of the COPD and are present in majority of the COPD patients. In patients with COPD emphysema was associated with rapid deterioration of lung function. Previous research reported that emphysema was associated with rapid deterioration of lung function in severe COPD patients^16^. Another research showed that presence of emphysema in COPD patients was associated with higher decline in FEV1 irrespective of the severity of disease^34^. Thus, assessment of emphysema should be included in routine clinical practice to identify patients at increased risk of lung function decline.

Exacerbation causes deterioration of COPD symptoms increase the risk of mortality and is associated with substantial economic burden on healthcare system^35–37^. Thus, prevention of exacerbation is a key goal in the management of COPD^6^. Impact of exacerbation frequency and hospital admission days was observed on rapid deterioration of lung function. For exacerbation frequency a significant difference was observed on rapid deterioration of lung function in patients suffering from 3 or more exacerbations during study period. Our findings are in line with the current literature. Previous studies demonstrated that frequent exacerbations cause rapid deterioration of lung function and exercise capacity^38^. Severe exacerbation requires long hospital stay. COPD patients who spent more than 8 days admitted in hospital showed rapid deterioration of lung function. This in in line with the previous research, which reported that each severe exacerbation event was associated with 87 ml/yr decline in FEV1 in COPD patients^39^.

Impact of comorbidities on rapid progression of disease was observed for CCI score. CCI score ≥ 3 was significantly associated with rapid deterioration of lung function. COPD is caused by inflammation of lungs. The drainage of inflammatory mediators from respiratory system into systemic circulation may trigger or worsen the effect of different comorbid conditions, such as peripheral vascular disease, hypertension, osteoporosis, diabetes and depression, which in turn results in worsening of COPD^40,41^. In a previous research coronary heart disease and peripheral vascular disease in addition to depression and anxiety were reported to have substantial effect on health status of COPD patients^42^. This shows a substantial relation among comorbidities and health status of COPD patients. Thus management of comorbid conditions can help to limit the disease in acute stage, reduce mortality and improve health status.

The 6MWT is a validated test to measure exercise capacity in COPD patients. Impaired exercise capacity is a well-established predictor of impaired lung function, exacerbation and mortality in COPD patients. Patients with mean walking distance < 350 were at increased risk of lung function deterioration. Exercise capacity decrease with the progression of disease. According to Celli reduced exercise capacity is the most reliable predictor of disease progression and should be considered in prognosis or worsening of COPD overtime^43^. Pinto-Plata et al. found that consistent decline in 6MWT was reliable predictor of COPD worsening over time and increased risk of mortality^44^. Thus, 6MWT can be used to assess the risk of rapid progression of disease over time.

In recent research inflammatory biomarkers are getting importance to categorize COPD patients and predict different outcomes like exacerbation and disease progression. Presence of high percentage of eosinophil cells in small airways was associated with increased risk of rapid decline in FEV_1_. Continuous cough, damage of airways or bacterial infection may increase the release of eosinophil from bronchial glands into the lumen^9^. Previous studies also reported that higher eosinophil count was associated with deterioration of lung function over time^45^. Our study strengthens this evidence.

This study may have few limitations. Patients reported outcomes may involve recall biasness, which can result in underreporting or over-reporting of an outcome. Biasness was reduced by cross matching the answers with patient medical files. Another limitation of the study was relatively small follow-up period of one year and sample sizes for subgroup analysis. Number of patients in rapid decline group were relatively small, which can affect the statistical power of the study to find risk factors for rapid deterioration of lung function. But despite few limitations, this study has several strengths. To ensure generalizability all eligible patients were included and no instructions regarding treatment were given to the healthcare professionals and treatment was decided solely by patient’s physician. Detailed characterization of the rapid decline group was performed. For comorbidities CCI score was used which gives better understanding of the impact of comorbidities according to severity and avoid underestimation of the impact of comorbidities. Most of the identified factors i.e. health status, symptoms, and occupational hazards were modifiable. Knowledge about these modifiable factors can ensure optimal monitoring of specific patients and identifying specific subgroups that may benefit from future novel therapies. Finding the risk factors to trigger disease progression can also help the health care practitioners and policy makers to limit disease in acute stage by taking in time measures. Moreover, during study period 32 (8.71%) patients showed no change in FEV_1_ value. This shows that lung function decline in COPD patients can be inhibited for longer period of time. Future investigations should focus on finding the factors which can be helpful in inhibiting the deterioration of lung function in COPD patients over a longer period of time. Emphasis should be placed on factors that can be easily adopted in clinical practice.

## Conclusions

In COPD management multidimensional assessment is an established strategy for better assessment of patients. During the study period mean annual decline in FEV1 was observed as 27.35 ml, whereas, 30.27% patients showed ≥ 60 ml decline in FEV1 value. Among different factors increased symptoms load, CCI score ≥ 3, abrupt decline in health status, exacerbation frequency ≥ 3, hospital admission days ≥ 8, chronic bronchitis and emphysema were reported as risk factors for rapid deterioration of lung function. Most of the identified factors were modifiable and can be controlled through proper management. Thus assessment of above mentioned factors should be included in routine clinical practice to identify patients at increased risk of lung function decline.

## Data Availability

Data is available on request from corresponding author.
